# Surveillance Snapshot: Mid-Season Influenza Vaccine Effectiveness Against Laboratory-Confirmed Ambulatory Influenza Among U.S. Active Component Service Members, December 1, 2024–February 8, 2025

**Published:** 2025-03-20

**Authors:** Angelia A. Eick-Cost, Zheng Hu

**Affiliations:** 1 Epidemiology and Analysis Branch, Armed Forces Health Surveillance Division, Public Health Directorate, Defense Health Agency, Silver Spring, MD

This Surveillance Snapshot provides an overview of the 2024-2025 mid-season analysis of influenza vaccine effectiveness (VE) against medically-attended ambulatory influenza infections among active component U.S. service members.

A case test-negative study design was implemented among the population of active component service members (ACSMs) from all services who were tested for influenza between December 1, 2024 and February 8, 2025—the period of peak influenza activity for the season. Data from the Defense Medical Surveillance System and standardized laboratory data provided by the Defense Centers for Public Health–Portsmouth were used for this analysis.^1^ Cases were defined as individuals with a positive influenza result from a rapid antigen, reverse transcription polymerase chain reaction (RT-PCR), or culture influenza assay. Test-negative controls (TNCs) were individuals with a negative influenza result from a RT-PCR or culture influenza assay. Crude odds ratios (ORs) were calculated, and multivariate logistic regression was used to calculate adjusted ORs (adjusted for sex, age category, any influenza vaccination in the previous 5 years, and month of diagnosis) and 95% confidence intervals (CIs). Estimates of VE were defined as (1 – OR) x 100.

There were 4,677 cases (4,413 A any subtype, 389 A[H3N2], 163 A[H1N1]pdm09, 274 B any subtype) and 12,787 TNC. Ten cases were both influenza A- and influenza B-positive. VE varied by influenza type (Table). Adjusted VE estimates for all influenza types and subtypes reached statistical significance. The results indicate moderate mid-season protection against influenza A(H1N1)pdm09 (adjusted VE 41%; 95% CI, 14-60) and A(H3N2) (adjusted VE 50%; 95% CI, 36-60) and low mid-season protection against influenza A (any subtype) (adjusted VE 14%; 95% CI, 5-22) and influenza B (adjusted VE 33%; 95% CI, 9-50).

The results of this analysis show low to moderate mid-season protection of the 2024-2025 seasonal influenza vaccines against medically-attended influenza A and B infections that resulted in an ambulatory care visit among ACSMs. As these estimates were obtained during the middle of the influenza season, VE estimates and confidence intervals may change when data for the full season are available and the sample sizes increase.

## Figures and Tables

**Table:** Influenza Vaccine Effectiveness Against Medically Attended, Laboratory-Confirmed Ambulatory Influenza, Active Component U.S. Service Members, 2024–2025 Season

Influenza Type	Vaccinated	Cases		Test-Negative Controls		Crude VE (95% CI)	Adjusted VE^a^ (95% CI)
		No.	%	No.	%		
**Any type**
	Yes	3,883	83	10,532	82	-5 (-14, 4)	15 (7, 23)
	No	794	17	2,255	18	–	–
**A (any subtype)**
	Yes	3,680	83	10,532	82	-7 (-18, 2)	14 (5, 22)
	No	733	17	2,255	18	–	–
**A(H1N1)pdm09**
	Yes	126	77	10,532	82	27 (-5, 50)	41 (14, 60)
	No	37	23	2,255	18	–	–
**A(H3N2)**
	Yes	290	75	10,532	82	37 (21, 50)	50 (36, 60)
	No	99	25	2,255	18	–	–
**B**
	Yes	213	78	10,532	82	25 (0, 44)	33 (9, 50)
	No	61	22	2,255	18	–	–

Abbreviations: VE, vaccine effectiveness; CI, confidence interval.

^a^Adjusted for sex, age category, prior vaccination (any influenza vaccine in previous 5 years), month of diagnosis.

## AUTHORS' AFFILIATION

Epidemiology and Analysis Branch, Armed Forces Health Surveillance Division, Public Health Directorate, Defense Health Agency, Silver Spring, MD: Dr. Cost, Ms. Hu
